# White Cord Syndrome Causing Transient Tetraplegia After Posterior Decompression and Fusion

**DOI:** 10.31486/toj.19.0081

**Published:** 2020

**Authors:** Christopher D. Busack, Bernard E. Eagleton

**Affiliations:** Department of Anesthesiology, Tulane University School of Medicine, New Orleans, LA

**Keywords:** *Anesthesia*, *ischemia*, *neurophysiological monitoring*, *neurosurgery*, *perfusion*, *quadriplegia*

## Abstract

**Background:** New neurologic deficits after spine surgery occur in less than 1% of cases. A particularly rare complication is white cord syndrome, a neurologic deterioration in the absence of obvious perioperative injury with concurrent hyperintense signal change on T2-weighted magnetic resonance imaging. The pathophysiologic mechanism is hypothesized to be an ischemia-reperfusion injury after the decompression of a chronically ischemic cord.

**Case Report:** A 63-year-old male underwent posterior cervical decompression and fusion for severe cervical stenosis and myelopathy. During the procedure, intraoperative neurophysiologic monitoring signals were lost. The patient developed acute postoperative tetraplegia attributed to white cord syndrome. Motor and sensory deficits improved after intravenous dexamethasone and intensive physical therapy.

**Conclusion:** The pathophysiology of white cord syndrome is unclear, and intraoperative anesthetic management strategies to prevent this syndrome are unknown. This case serves to educate perioperative physicians to suspect this rare syndrome, encourage research into its pathophysiology, and guide clinicians in formulating therapeutic regimens.

## INTRODUCTION

New neurologic deficits are rare complications following spine surgery, occurring at a rate less than 1%.^1,2^ The rate may be higher in specific patients with severe spinal stenosis, as one review of 114 patients demonstrated a complication rate of nearly 8%.^3^ A retrospective review of 11,817 patients with new deficits revealed epidural hematoma, inadequate decompression, and acute ischemia to be the most common causes of neurologic complications, with incidences of 38%, 23%, and 19%, respectively.^1^ The routine use of intraoperative neurophysiologic monitoring (IONM) detects neurologic deficits with >90% sensitivity, allowing perioperative physicians to alter management to resolve the insult.

Recent case reports (published from 2013 to 2018) describe a novel pathophysiologic phenomenon called white cord syndrome (Table),^4-7^ neurologic deterioration with concurrent hyperintense signal change on T2-weighted magnetic resonance imaging (MRI) in the absence of obvious perioperative complications. T2 hyperintensity is a nonspecific finding but indicates an increase in water content in the area, as seen with acute ischemia or demyelination.^8^ The pathophysiologic mechanism of white cord syndrome is hypothesized to be ischemia-reperfusion injury via damaging oxygen-derived free radicals following the acute reperfusion of chronically ischemic tissue.^4,6^

**Table .** Published Reports of White Cord Syndrome

Case Report	Preoperative Deficit	Surgical Case	Postoperative Deficit	Intervention
Chin et al, 2013^4^	Radiculomyelopathic pain, ataxia, Nurick score 3	C4-C5, C5-C6 anterior cervical discectomy and fusion	C6 incomplete tetraplegia, new left-sided weakness	Hydrocortisone, inpatient rehabilitation, Nurick score 4
Giammalva et al, 2017^5^	Radiculomyelopathic pain, gait disturbance, urinary incontinence, spastic tetraparesis, Nurick score 3	C3-C4, C5-C6 anterior cervical discectomy and fusion	Tetraparesis with complete paraplegia, motor weakness to upper limbs with diffuse spastic hypertonia	Methylprednisolone, inpatient rehabilitation, Nurick score 4
Antwi et al, 2018^6^	Intermittent paresthesias, numbness, balance difficulties, Nurick score 1	C4-C7 posterior decompression via laminectomy, C3-C7 instrumented arthrodesis	Left-sided hemiparesis	Methylprednisolone, dexamethasone taper, inpatient rehabilitation, Nurick score 4
Vinodh et al, 2018^7^	Paraparesis, acute urinary retention	C2-C5 posterior laminectomy C1-C2, C5-C6 fusion	C3 quadriplegia	Dexamethasone, palliative care

To our knowledge, this report is the fifth published case of a patient who developed white cord syndrome.

## CASE REPORT

**Figure 1.**
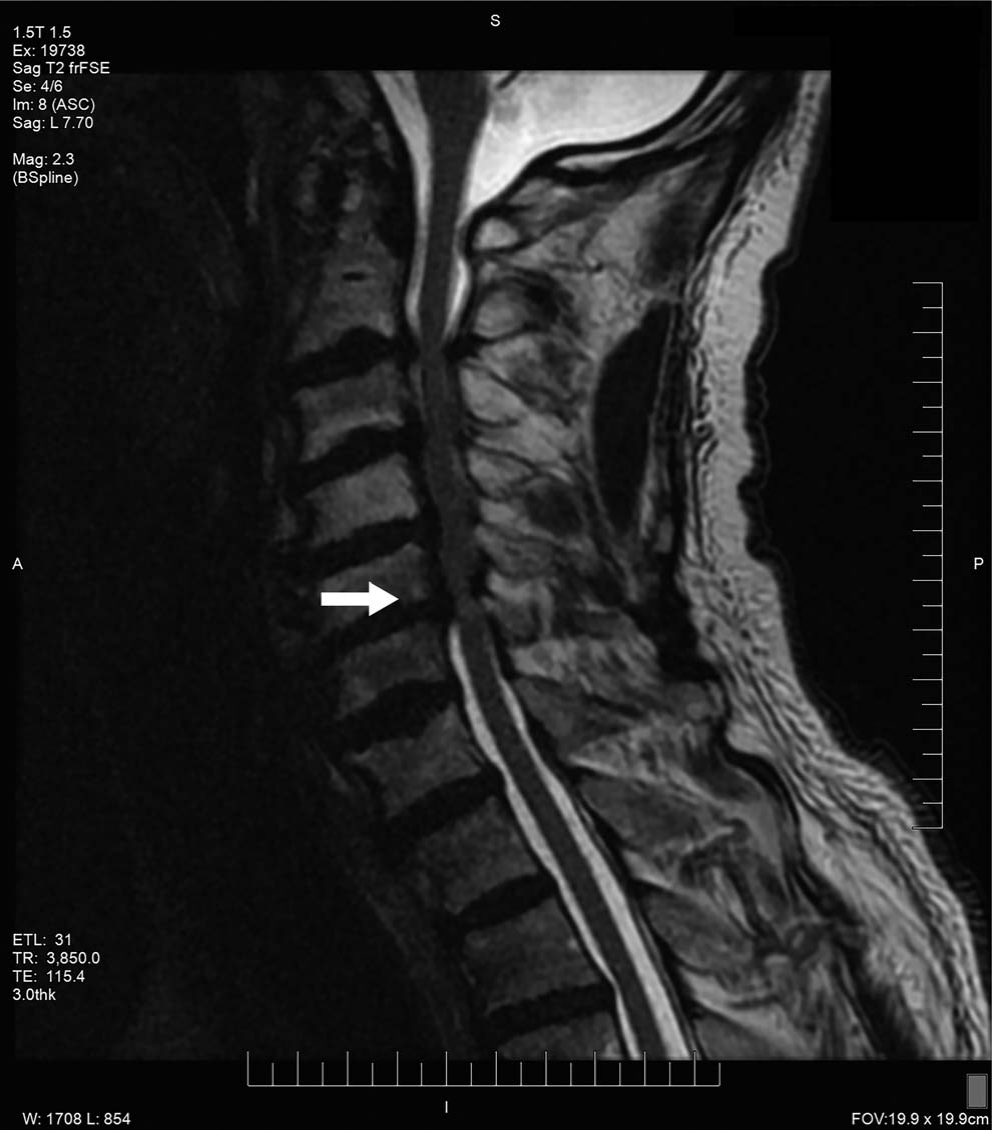
**Preoperative T2-weighted magnetic resonance sagittal image of 63-year-old-male with severe cervical spinal stenosis demonstrates myelopathy with associated hyperintensity at the C5-C6 level (arrow).**

A 63-year-old male with a 1-year history of progressive difficulty walking, declining balance, and neuropathic pain in his extremities (Nurick grade 3, modified Japanese Orthopaedic Association [mJOA] score of 15) presented to the preoperative testing clinic in preparation for a C3-C6 laminectomy and C2-T1 posterior fusion. Cervical MRI demonstrated severe cervical stenosis from C2-C3 to C5-C6 with associated hyperintense intramedullary signal change at C5-C6 (Figure 1).

The patient's medical history was notable for a 15 pack-year smoking history and poorly controlled hypertension. He took metoprolol tartrate 25 mg twice daily and oxycodone 20 mg twice daily. His surgical history included a remote lumbar transforaminal interbody fusion in 2001. The patient's temperature was 36°C, blood pressure was 189/96 mmHg, and heart rate was 54 bpm. Physical examination was notable for decreased cervical range of motion because of pain. The patient exhibited 5/5 strength in all muscle groups of the upper and lower extremities except for 4/5 strength in bilateral biceps and triceps. Sensation to light touch was decreased in bilateral upper and lower extremities. Bilateral upper and lower extremities revealed hyperreflexia. The patient's laboratory values were notable for hemoglobin of 15 g/dL.

On the morning of surgery, the patient's blood pressure was 159/90 mmHg (mean arterial pressure [MAP] of 113 mmHg), and his heart rate was 64 bpm. In the operating room, the patient was positioned in neutral cervical position, and lack of symptoms was confirmed in this position prior to administration of induction medications. General anesthesia was induced with propofol, fentanyl, lidocaine, ketamine, and succinylcholine. Gentle bag mask ventilation was achieved during maintenance of manual inline stabilization. Video-assisted laryngoscopy (Storz C-MAC) obtained a Cormack-Lehane grade 1 view, and endotracheal intubation was secured. General anesthesia was maintained with dexmedetomidine (0.1 mcg/kg/h), propofol (100 mcg/kg/min), and inhaled sevoflurane (1%). The patient underwent C3-C6 posterior decompression via laminectomy and C2-T1 instrumented fusion. The spinal cord and exiting nerve roots were decompressed via total foraminotomies. Bispectral index, somatosensory evoked potentials (SSEPs), and motor evoked potentials (MEPs) were monitored throughout the procedure. The MAP goal of >85 mmHg was achieved with ephedrine and phenylephrine. Systolic blood pressure >200 mmHg was treated with fentanyl and nicardipine.

**Figure 2.**
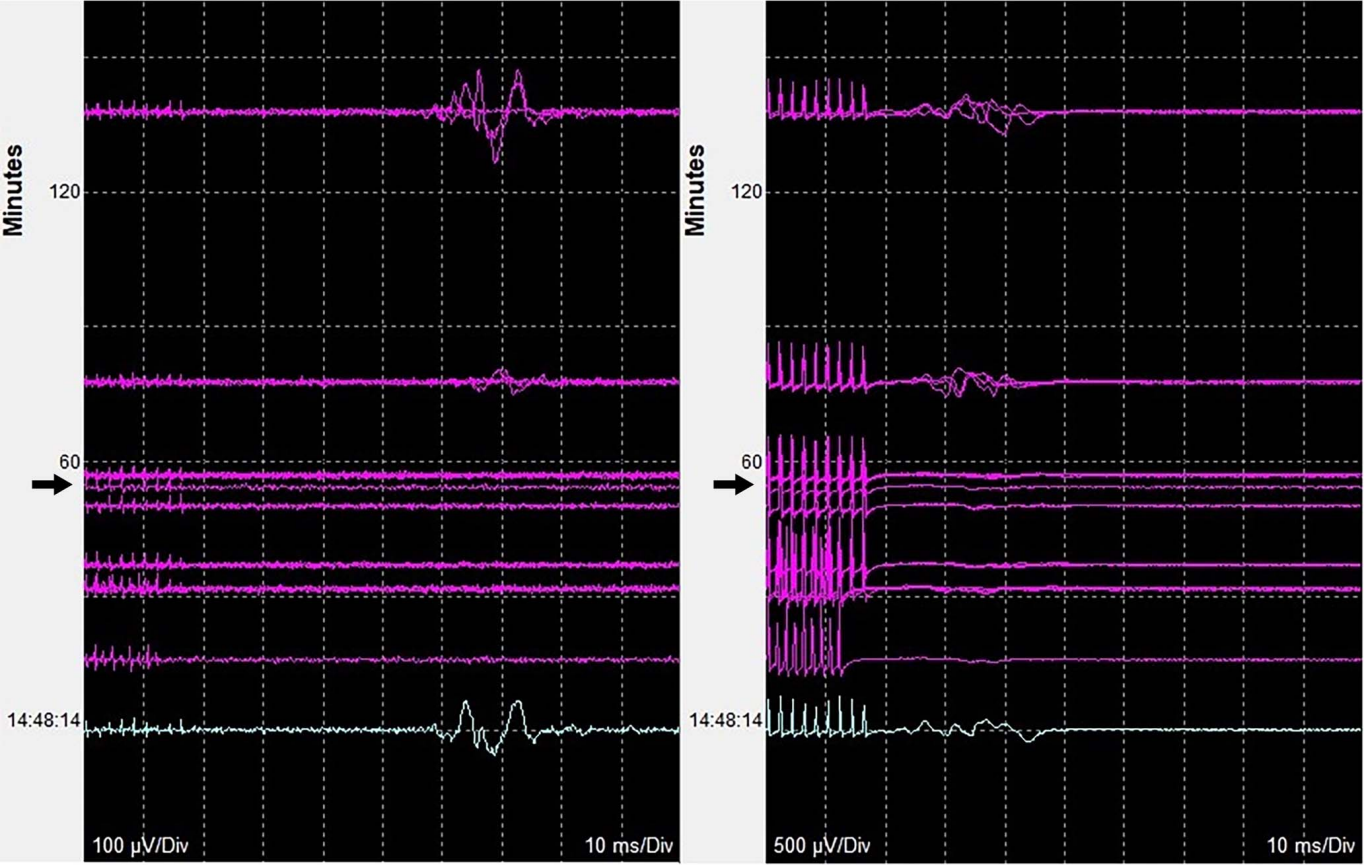
**Intraoperative neurophysiologic monitoring for left triceps (left) and left extensor hallucis brevis-abductor hallucis muscles (right) demonstrates baseline motor evoked potentials (top and repeated for reference purpose at bottom) and subsequent loss of signals (arrows) approximately 90 minutes after incision.**

Ninety minutes after incision, when the laminectomies were completed, the neurophysiologist noted the abrupt loss of MEPs and diminished SSEPs in the bilateral upper and lower extremities (Figure 2). Hardware had not yet been placed, and the cervical spinal cord was fully decompressed. MAP was 115 mmHg, and no change had been made to the anesthetic regimen. The patient's MAP had been maintained at 85 to 133 mmHg throughout the procedure. His temperature was 35.6°C, and estimated blood loss at the time of signal loss was 100 mL. The cord was normal in appearance but was noted to expand dramatically. No cerebrospinal fluid (CSF) leak or hemorrhage was noted. Noting the sudden cord expansion and edema, the surgeon suspected a diagnosis of white cord syndrome. The patient was given intravenous (IV) dexamethasone 8 mg in the operating room (he had received 4 mg of dexamethasone preoperatively for postoperative nausea prophylaxis).

The C2-T1 fusion was completed, but MEPs and SSEPs never improved during the remainder of the procedure. The patient was extubated and transferred to the neurosurgical intensive care unit. His initial physical examination demonstrated lack of sensation below the T3 level and 0/5 motor strength of all muscle groups except 4/5 strength of bilateral deltoids. Postoperative hemoglobin was 13.9 g/dL.

During his hospitalization, the patient improved with IV dexamethasone therapy. He received 8 mg every 6 hours for the first 48 hours, followed by 4 mg every 6 hours for the next 72 hours. Oral dexamethasone was then tapered for 2 weeks. The patient's neurologic examination on the date of discharge revealed a T10 sensory level and 2/5 motor strength of bilateral lower extremities with the exception of 3/5 motor strength of bilateral knee extension and hip flexion. His upper extremities had returned to 5/5 strength with the exception of 4/5 in grip strength bilaterally.

The patient was discharged to an inpatient rehabilitation center on postoperative day 10 where he completed 3 hours of physical therapy daily for 4 weeks. The patient was discharged from inpatient rehabilitation on postoperative day 30 with bilateral upper and lower extremity strength of 5/5 with the exception of 4/5 bilateral dorsiflexion (Nurick grade 4, mJOA score of 13). He then completed 8 weeks of home and outpatient physical therapy. A postoperative MRI was not performed.

## DISCUSSION

Chin et al first described white cord syndrome in 2013, noting the white appearance of the spinal cord edema observed on postoperative T2-weighted MRI.^4^ The pathophysiology of white cord syndrome is hypothesized to be attributable to oxygen-derived free radical damage after rapid cord expansion and reperfusion.^4,6^ In a chronically compressed ischemic spinal cord, the blood–spinal cord barrier is disrupted, creating an area of spinal cord that is vulnerable to reperfusion injury upon decompression. Chronic compression creates an endothelial milieu prone to damage by oxygen-derived free radicals, causing loss of capillary contact with astrocyte foot processes.^4,9^ The disrupted blood–spinal cord interface allows for tumor necrosis factor-α migration and apoptotic pathway activation.^4,9^ This pathophysiologic mechanism has been simulated in mouse models.^10^ In mice with severely compressed spinal cords, oxidative stress indicators increased after decompression. In contrast, mice with mildly compressed cords did not exhibit these indicators after reperfusion.

Taher and colleagues have proposed neuropraxia attributable to recoil and change in the cross-sectional dimension of the cord as an etiology.^11^ The pathogenesis of white cord syndrome warrants investigation, with mechanical, vascular, and biochemical factors all playing some role.

Regardless of the true pathophysiologic mechanism, suspected cases of white cord syndrome warrant collaborative efforts between surgeons and anesthesiologists to avoid poor neurologic outcomes. Anesthesiologists manage blood pressure intraoperatively, and the hemodynamics surrounding the development of white cord syndrome warrants rethinking of blood pressure goals. Spinal cord perfusion mimics the perfusion of the brain because spinal cord perfusion depends upon the difference between MAP and CSF pressure. The autoregulatory zone of stable cerebral perfusion is classically described as a range from 70 to 150 mmHg. However, this range is likely higher in patients with chronic hypertension or spinal cord ischemia, meaning that these patients require a higher MAP intraoperatively to stay within the autoregulatory zone. *Miller's Anesthesia* recommends maintaining MAP within 10% of the patient's baseline MAP for patients with acute spinal cord injury, although stricter control may be required in patients with myelopathy or trauma to the spine.^12^ Various sources cite MAP goals of 80 to 85 mmHg for spine surgery,^12-15^ yet this value seems to have been selected based on traumatic spinal cord injury studies.^16^ This MAP goal was originally chosen arbitrarily, and its benefit has been debated.^17^ A 2017 systematic review of 9 separate case series of patients with spinal cord injuries concluded that a MAP goal of 85 to 90 mmHg for 5 to 7 days after injury should be considered, given the limitation that most studies are retrospective and have a low sample size.^13^ Despite the lack of high-quality evidence, the American Association of Neurological Surgeons and Congress of Neurological Surgeons joint guidelines recommend maintaining MAP of at least 85 mmHg for patients with spinal cord injuries.^15^ Even less evidence is available to guide intraoperative blood pressure management for spinal surgery, so the spinal cord injury recommendations have been applied to the operating room.

IONM has become the standard of care for spinal surgery. Loss of intraoperative signals is indicative of direct cord injury, compression, or ischemia. In a 2018 series of 452 pediatric scoliosis repairs, Yang et al showed that raising MAP alone was curative in restoring 20% of lost IONM signals.^18^ Anecdotal evidence from practicing neurophysiologists concurs with this finding that blood pressure alteration often improves intraoperative signals. Our patient's MAP was lowest after induction of anesthesia, reaching a nadir of 85 mmHg, yet IONM signals remained strong for the first 90 minutes of the procedure. At the time of decompression, MAP was 115 mmHg, raising the question of what the MAP value for appropriate spinal cord perfusion should have been. Perhaps this MAP was actually too high. Given that the suspected pathophysiology of white cord syndrome is a reperfusion phenomenon, perhaps a high MAP delivers too much mechanical flow and too high an oxygen-derived free radical load to the cord. Studies have shown that hypertension following mechanical thrombectomy for large vessel occlusions increases complications and worsens outcomes,^19,20^ which has led many centers to maintain systolic blood pressure <140 mmHg after successful recannulization.^21^ In the same way that blood pressure targets are decreased after thrombectomy of a large vessel occlusion due to open reperfusion,^19^ reduction of the MAP target after decompression of a chronically compressed cord may reduce cord edema and reperfusion injury. Future studies for spinal surgeries should investigate not only minimum MAP goals but should also include maximum MAP goals to better guide providers in the neurosurgical care team.

CSF pressure may also be a factor to consider in the management of white cord syndrome. At the site of a newly decompressed spinal cord, CSF pressure is increased, and this finding is corroborated by surgeons in real time with visual bulging of the thecal sac intraoperatively.^11^

Because spinal cord perfusion is dependent on CSF pressure, preoperative placement of a CSF drain may aid in anesthetic management of a patient with a severely compressed spinal cord. Elevated CSF pressures inhibit perfusion to the edematous cord, so gradually reducing CSF pressure prior to complete decompression, if we had considered it, may have mitigated cord edema in our patient.

Although clinical experience and animal models imply that the severity of compression corresponds with the degree of neurologic deficit,^10^ studies attempting to correlate the degree of T2-weighted signal change with neurologic outcome have been inconclusive.^22^

## CONCLUSION

Case numbers are insufficient to determine particular risk factors for white cord syndrome. High preoperative T2 signal intensity has been a consistent finding in all published case reports, including ours. Studies are needed to clarify risk factors, characterize the pathophysiology, and determine the appropriate perioperative management of this rare syndrome.

## Notes

This article meets the Accreditation Council for Graduate Medical Education and the American Board of Medical Specialties Maintenance of Certification competencies for Patient Care and Medical Knowledge.
